# Uncovering negative results: Introducing an open access journal “Journal of Pharmaceutical Negative Results”

**DOI:** 10.4103/0976-500X.72364

**Published:** 2010

**Authors:** Vipra Kundoor, K. K. Mueen Ahmed

**Affiliations:** *U. S. Food and Drug Administration, Rockville, Maryland, USA*; 1 *Department of Pharm Sciences, College of Clinical Pharmacy, King Faisal University, Al-Ahsa, Saudi Arabia*

“Once you replace negative thoughts with positive ones, you’ll start having positive results.”

*Willie Nelson*

Drug discovery nowadays is a highly innovative process by which new drugs are discovered and/or designed. It is expected that in the year 2010, at least 50% of all new approved drugs will come from the biopharmaceutical sector. The discovery and validation process is mostly carried out by new innovative and medium-sized companies. But despite advances in technology and greater understanding of biological systems, drug discovery is still a lengthy process. Specialized drug discovery companies need an ideal business climate, that is, political, economic and social conditions, to be able to cope with the entrepreneurial risks of drug development.

Global Pharmaceutical Industry is at the crossroads. On one side, market demand for novel and better medicines is getting redefined and growing due to demographic, economic and epidemiologic trends, but at the same time, pharmaceutical companies are finding it difficult to survive since they are unable to innovate and discover new molecules. The pipeline of the new drug molecules is drying fast due to poor R&D productivity, high failure rate and generic competition.[1]

Scientific research proceeds with an infinite variety in the availability of data, the maturity of conceptions and the mix of conviction and uncertainty. Often a major discovery is the culmination of a process with numerous blunders, wrong turns, false hypotheses, missed opportunities, persistent hard work, lucky breaks, rational arguments, insightful conjectures and accumulating knowledge over decades. But none of the failures are being reported or published in the journals and other resources. This part is always ignored because of the publishing style of most of the journals in the pharmaceutical field.

Welcome to the *Journal of Pharmaceutical Negative Results (JPNR)*. We are proud to introduce you to a new home for negative results focused on all aspects of Pharmacy. *Journal of Pharmaceutical Negative Results*[2] represents the first open access source for research concerning negative results and will be a valuable resource for researchers all over the world, both to those who are already experts and also to those entering the field.

*Journal of Pharmaceutical Negative Results* is a peer-reviewed journal developed to publish original, innovative and novel research articles resulting in negative results. This peer-reviewed scientific journal publishes theoretical and empirical papers that report negative findings and research failures in the pharmaceutical field. Submissions should have a negative focus, which means the output of research yielded negative results is being given more preference. All theoretical and methodologic perspectives are welcome. We also encourage the submission of short papers/communications presenting counterexamples to usually accepted conjectures or to published papers.[2,3]

**Figure 1:**
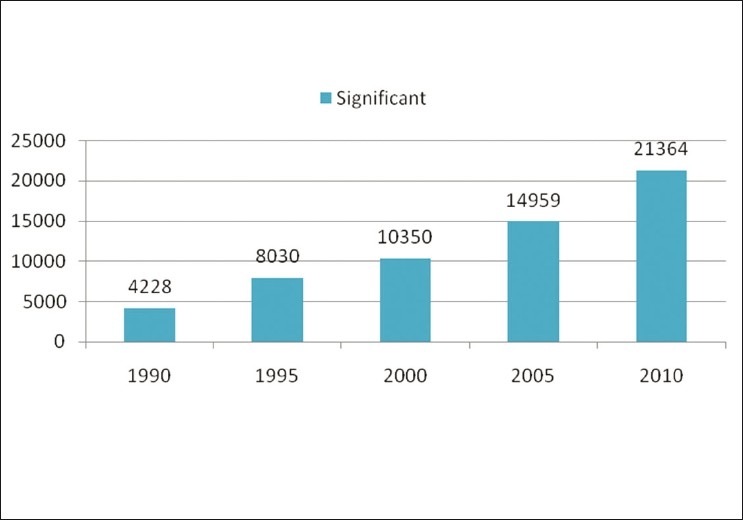
The number of PubMed query results (Y-axis) with the year limits (X-axis) for the terms for MESH terms “Significant, Pharmaceutical”

**Figure 2:**
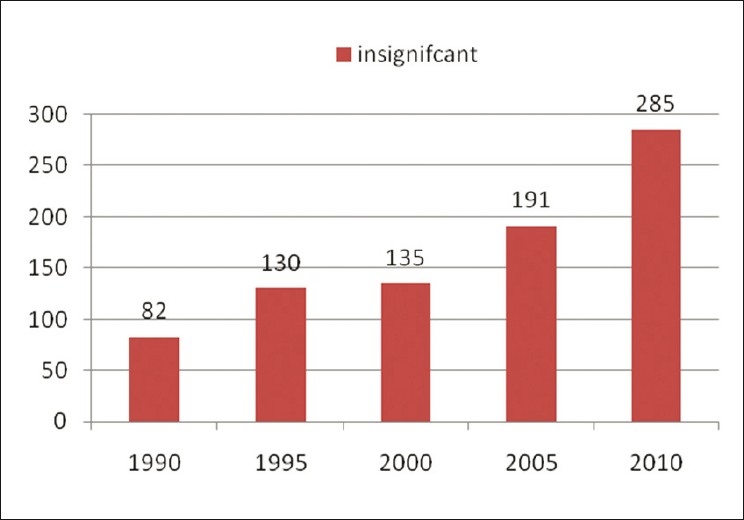
The number of pubmed query results (Y-axis) with the year limits (X-axis) for the terms for MESH terms “Insignificant, Pharmaceutical”

The main aim of the journal is to publish negative results, so that the newer generation of researchers should not waste their time and money repeating the same studies and finding the same unpublishable results. Most of the journals publish only positive results and findings, however, they do not publish negative results. We believe that negative results are also a part of quality research. Thus, the journal’s focus is not limited to negative issues but will also address the advances in pharmaceutical research, including failures. This Journal will provide multilateral coverage of numerous applied and basic aspects of current pharmaceutical research[4] [Figures 1 and 2].

Taking into account the growing amount of pharmaceutical journals available, one can argue whether we need another one. However, any scientist working on theoretical and practical aspects of pharmaceutical field would undoubtedly agree that there are few or no journals covering or boldly accepting negative results and that there are no journals providing immediate open access. We performed an informal analysis [Figures 1 and 2] that shows the consistent interest of researchers to publish their original data in peer-reviewed pharmacy journals. A simple PubMed query resulted in more than 58,931 for significant reports and just 823 for insignificant results. However, with the large proportion of articles (over 90%) related to pharmaceutical field with positive results being published, it looks like research is always successful—is this the real scenario? Therefore, numerous scientists all over the world, especially those in the developing countries, miss the opportunity to keep abreast with the failures in Pharmaceutical research. Since there are no open access journals in the pharmaceutical field covering negative results, the present journal fills this gap in the current pharmaceutical publishing.

*Journal of Pharmaceutical Negative Results* aims to become a significant international participant in this field. The purpose of the journal is, therefore, to publish timely and high-quality articles [See author information at http://www.pnrjournal.com/contributors.asp] concerning failures in techniques and negative or unexpected results in all areas of pharmacy. To achieve this, the Editorial Board of PNR Journal gathers well-known experts from all over the world.[4] To make a decision whether a manuscript fits the format of publishing in the journal, the Editor-in-Chief or the corresponding specialists from the Editorial Board will immediately screen a submission and the appropriate manuscripts will be sent for peer review to two independent reviewers. Based on their reports and scoring, a final decision will be made regarding the acceptance of manuscripts. The Editor-in-Chief reserves the right to make final decisions concerning each manuscript submitted to the journal. At first sight, our peer-review system does not significantly differ from that proposed by other journals. However, we encourage our Editorial Board members as well as reviewers to be motivated critical advisers for improving a manuscript rather than suggesting rejection, which is unfortunately a common experience for anyone who has ever tried to publish his or her original negative research results.

*Journal of Pharmaceutical Negative Results* is published by the open access publisher, Medknow Publications and Media Pvt. Ltd, India.[5] When submitting a manuscript, authors will be required to declare competing interests. In addition, for all the manuscripts accepted for publication, an article-processing charge will be levied, to cover the costs incurred by open access publication. We believe that the authors’ ability to publish their negative research should be governed only by the quality of the work and not financial limitations.

Considering all the advantages of this journal, we believe this journal to be of great benefit to the field of pharmacy. We invite you to work together with us to create this new and high-visibility open access forum for the pharmaceutical sciences.
